# Fatal Seatbelt Syndrome in an Elderly Wheelchair User: A Case Report

**DOI:** 10.7759/cureus.80046

**Published:** 2025-03-04

**Authors:** Yuki Sugitani, Akihiro Yashio, Ayumu Kuwahara, Hiroya Chiba, Masahito Hitosugi

**Affiliations:** 1 Department of Emergency Medicine, Otsu City Hospital, Otsu, JPN; 2 Department of Legal Medicine, Shiga University of Medical Science, Otsu, JPN

**Keywords:** blunt abdominal trauma, geriatric trauma, motor vehicle accident, ohca, out-of-hospital cardiac arrest, reboa, resuscitative endovascular balloon occlusion of the aorta, seat belt syndrome, traffic safety, wheelchair user

## Abstract

Elderly wheelchair users face unique challenges in traffic collisions due to physiological fragility and the limitations of standard vehicle seatbelt systems. Seatbelt syndrome, a pattern of abdominal organ and spinal injuries, can be severe when seatbelts do not properly align with an individual's body configuration. Moreover, geriatric out-of-hospital cardiac arrest (OHCA) has a low survival rate, even with advanced prehospital care.

This case study presents a 90-year-old woman, secured in her wheelchair with a two-point lap belt, who was involved in a low-speed rear-end collision. Initially alert, she rapidly deteriorated into shock and subsequently exhibited pulseless electrical activity (PEA), ultimately leading to her demise despite attempts at resuscitation using resuscitative endovascular balloon occlusion of the aorta (REBOA) and emergency thoracotomy. Postmortem examination revealed hepatic and splenic injuries consistent with seatbelt syndrome.

The patient's advanced age, short stature, and wheelchair dependence likely contributed to excessive abdominal force and fatal hemorrhage. While REBOA can provide transient circulatory stabilization, prolonged full occlusion increases the risk of ischemic complications. Emergency thoracotomy also shows limited benefit in geriatric blunt trauma, highlighting the need for more selective criteria.

This case underscores the importance of improving seatbelt restraint systems for wheelchair users, refining guidelines for REBOA and emergency thoracotomy in geriatric trauma, and implementing multifaceted prevention strategies to reduce avoidable deaths among elderly wheelchair users.

## Introduction

In geriatric trauma, physiological vulnerability, multiple comorbidities, and skeletal fragility are well-known risk factors that lead to markedly poor outcomes, particularly in cases of out-of-hospital cardiac arrest (OHCA) [1,2]. Because older adults often lack sufficient physiological reserve to cope with severe injuries or prolonged shock, even relatively minor trauma can precipitate life-threatening complications.

Elderly patients who rely on a wheelchair face additional challenges associated with suboptimal seatbelt fit, which can significantly increase the risk of seatbelt syndrome, a constellation of abdominal organ, spinal, and vascular injuries [3-5].

We present the case of a 90-year-old woman (height 140 cm), seated in a wheelchair and restrained only by a two-point lap belt. She sustained fatal blunt abdominal trauma in a low-speed collision. Aggressive interventions, including resuscitative endovascular balloon occlusion of the aorta (REBOA) and emergency thoracotomy (ET), were attempted but ultimately proved unsuccessful.

Specifically, this report addresses the following four key points: (1) REBOA and ET in geriatric blunt trauma, (2) the low survival rate of geriatric traumatic OHCA and the importance of accident prevention, (3) mechanisms and risk factors underlying seatbelt syndrome, and (4) unique seatbelt fit challenges for wheelchair users. By examining these points, we aim to highlight the critical need for improved restraint systems, refined treatment guidelines, and multifaceted prevention strategies to reduce mortality in this growing vulnerable population.

In particular, wheelchair users may be at a heightened risk of seatbelt syndrome due to improper belt positioning, which can lead to severe intra-abdominal or spinal injuries in even low-speed collisions.

## Case presentation

Patient background

A 90-year-old woman (height 140 cm, weight 40 kg) with a past medical history of left femoral neck fracture, urinary tract infection, and hypertension customarily relied on a wheelchair for mobility.

Accident circumstances and immediate course

She was being transported in an adapted vehicle, with her wheelchair secured behind the driver's seat. A two-point lap belt was placed across her abdomen as the sole restraint mechanism. The accident occurred at 10:30 AM. While traveling at approximately 30 km/h, the vehicle rear-ended another car that was stopped at a traffic signal.

Initially, the patient was alert and reported feeling fine. However, she then rapidly deteriorated, becoming unresponsive and showing signs of shock, which prompted an emergency call to the local emergency medical services (EMS) at 10:42 AM.

Overview of the Traffic Accident

A kei car traveling at approximately 30-40 km/h collided with a standard passenger vehicle that was stopped at an intersection, waiting to make a right turn. The passenger vehicle sustained moderate rear-end damage, while the kei car incurred minor front-end damage. Neither vehicle's airbags deployed, and there was no airbag installed in the rear seat of the kei car. The driver of the rear-ended passenger vehicle suffered minor injuries, and the kei car's driver was unharmed.

EMS course

The patient's prehospital course is summarized in Table 1. The accident occurred at 10:30 AM, and EMS was notified at 10:42 AM, representing a 12-minute interval from crash to call. Upon EMS arrival (10:52), she was found to be in shock, with a blood pressure of 70/59 mmHg and Glasgow Coma Scale (GCS) score of 3 (E1, V1, M1). Although assisted ventilation was initiated promptly, the patient's condition deteriorated to pulseless electrical activity (PEA) at 11:07, necessitating cardiopulmonary resuscitation (CPR).

**Table 1:** EMS course EMS: emergency medical services; GCS: Glasgow Coma Scale; PEA: pulseless electrical activity; CPR: cardiopulmonary resuscitation; IV: intravenous; RR: respiratory rate; HR: heart rate; BP: blood pressure

Time	Event
10:30	Accident occurred
10:42	EMS contacted
10:52	EMS arrival: GCS 3, RR 12 breaths/min, HR 99 bpm, BP 70/59 mmHg
10:56	Assisted ventilation initiated
11:03	Transport commenced; sought shock-management guidance
11:07	PEA confirmed→CPR begun
11:13	Laryngeal tube inserted
11:17	IV access established
11:18	IV epinephrine 1 mg administered
11:19	Arrival at the receiving hospital

In-hospital course

Despite ongoing resuscitative efforts, her rhythm remained in PEA. On examination, there were no overt external hemorrhages or seatbelt marks. The Focused Assessment with Sonography for Trauma (FAST) revealed pericardial effusion and fluid around the spleen, suggesting liver and spleen injuries. The team proceeded with an ET, which showed clear pericardial fluid and effectively excluded cardiac tamponade. The patient's admission laboratory data are shown in Table 2. Notably, she had severe metabolic acidosis (pH 6.999), hyperkalemia (5.9 mEq/L), marked coagulopathy (activated partial thromboplastin time (APTT) >180 seconds, fibrinogen 34 mg/dL), and profound anemia (hemoglobin 6.1 g/dL). Intra-aortic balloon occlusion (IABO/REBOA) catheter was then inserted via the right femoral artery, and four units of packed red blood cells (PRBCs) were transfused. However, PEA persisted, and the patient was pronounced dead at 12:23 PM. Because the patient's hemodynamic status had deteriorated, performing a CT or contrast-enhanced CT was deemed difficult.

**Table 2:** Laboratory findings ABG: arterial blood gas; CBC: complete blood count; pH: hydrogen ion exponent; pCO_2_: partial pressure of carbon dioxide; pO_2_: partial pressure of oxygen; Na: sodium; K: potassium; Cl: chloride; Ca²⁺: calcium ion; HCO₃⁻: bicarbonate; WBC: white blood cell; RBC: red blood cell; APTT: activated partial thromboplastin time; CK: creatine kinase; AST: aspartate aminotransferase; ALT: alanine aminotransferase; LD (LDH): lactate dehydrogenase; γGTP: gamma-glutamyl transferase; ChE: cholinesterase; BUN: blood urea nitrogen; CRP: C-reactive protein

	Patient value	Reference range
ABG		
pH	6.999	7.35-7.45
pCO₂ (mmHg)	22.4	35-45
pO₂ (mmHg)	218.6	75-100
Na (mEq/L)	139	135-145
K (mEq/L)	5.9	3.5-5.0
Cl (mEq/L)	119	98-106
Ca²⁺ (mmol/L)	1.06	1.12-1.32
Glucose (mg/dL)	152	70-99
Lactate (mmol/L)	15.5	0.5-2.2
HCO₃⁻ (mEq/L)	5.4	22-29
CBC		
WBC (/µL)	9900	4500-11,000
RBC (×10⁶/µL)	1.97	4.2-5.4
Hemoglobin (g/dL)	6.1	12-15.5 (F), 13.5-17.5 (M)
Hematocrit (%)	20.3	37-47 (F), 42-52 (M)
Platelets (×10³/µL)	41	150-400
Coagulation		
APTT (seconds)	>180	30-40
Fibrinogen (mg/dL)	34	200-400
D-dimer (µg/mL)	399.6	<0.5
Others		
CK (U/L)	86	55-170
AST (U/L)	262	10-40
ALT (U/L)	168	7-56
LD (U/L)	755	122-222
γGTP (U/L)	5	9-48
ChE (U/L)	66	4.6-11.2
Total bilirubin (mg/dL)	0.2	0.1-1.2
Direct bilirubin (mg/dL)	0.1	0.0-0.3
Total protein (g/dL)	2.9	6.4-8.3
Albumin (g/dL)	1.3	3.5-5.0
BUN (mg/dL)	13	7-20
Creatinine (mg/dL)	0.58	0.6-1.2
CRP (mg/dL)	0.17	<0.5

Postmortem CT

**Figure 1:**
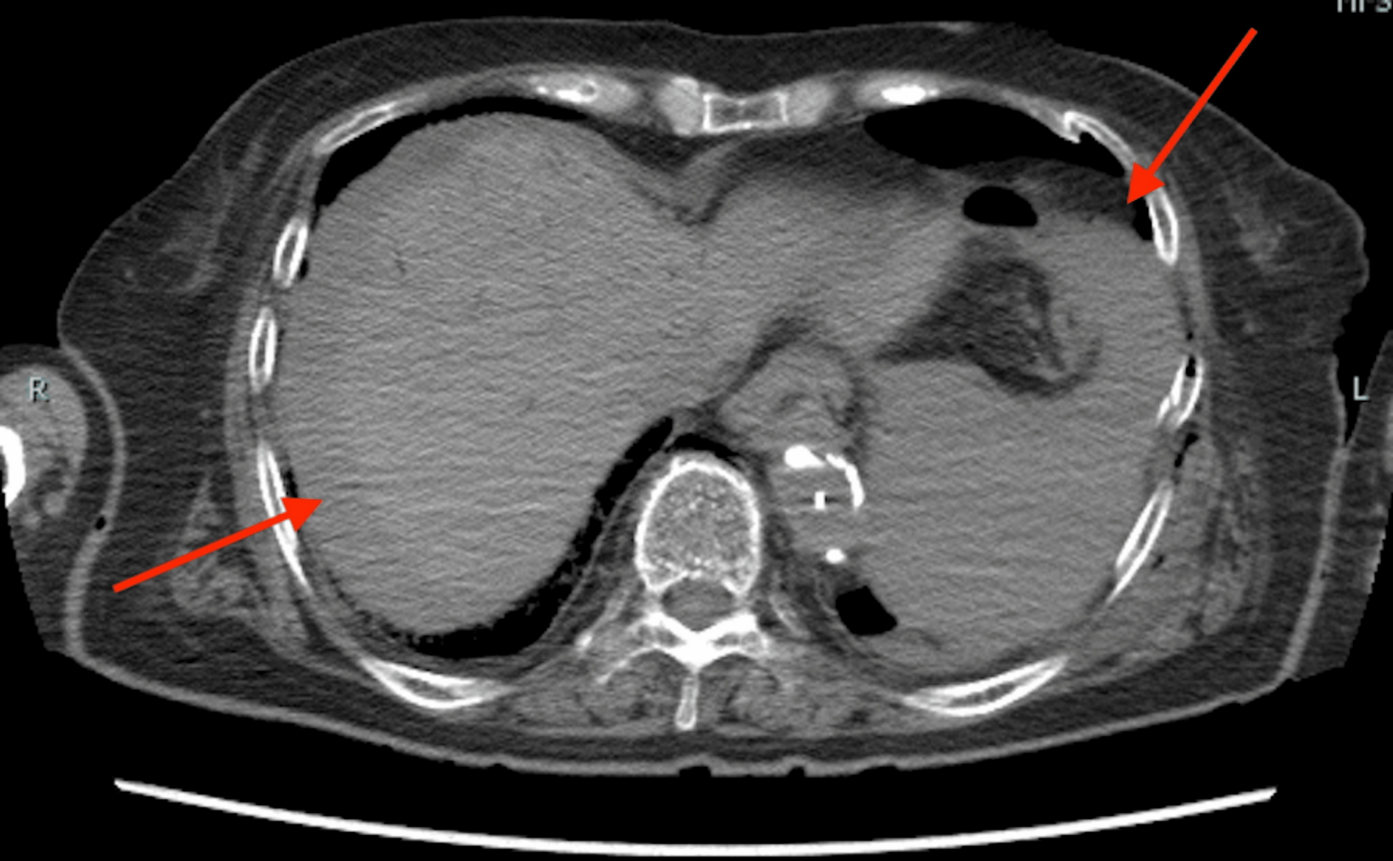
CT image (abdomen) On the CT scan, intraperitoneal hemorrhage was observed around the liver and spleen, suggesting possible hepatic and splenic injuries. The red arrows indicate areas around the liver and spleen where what appears to be bloody ascites is present.

**Figure 2:**
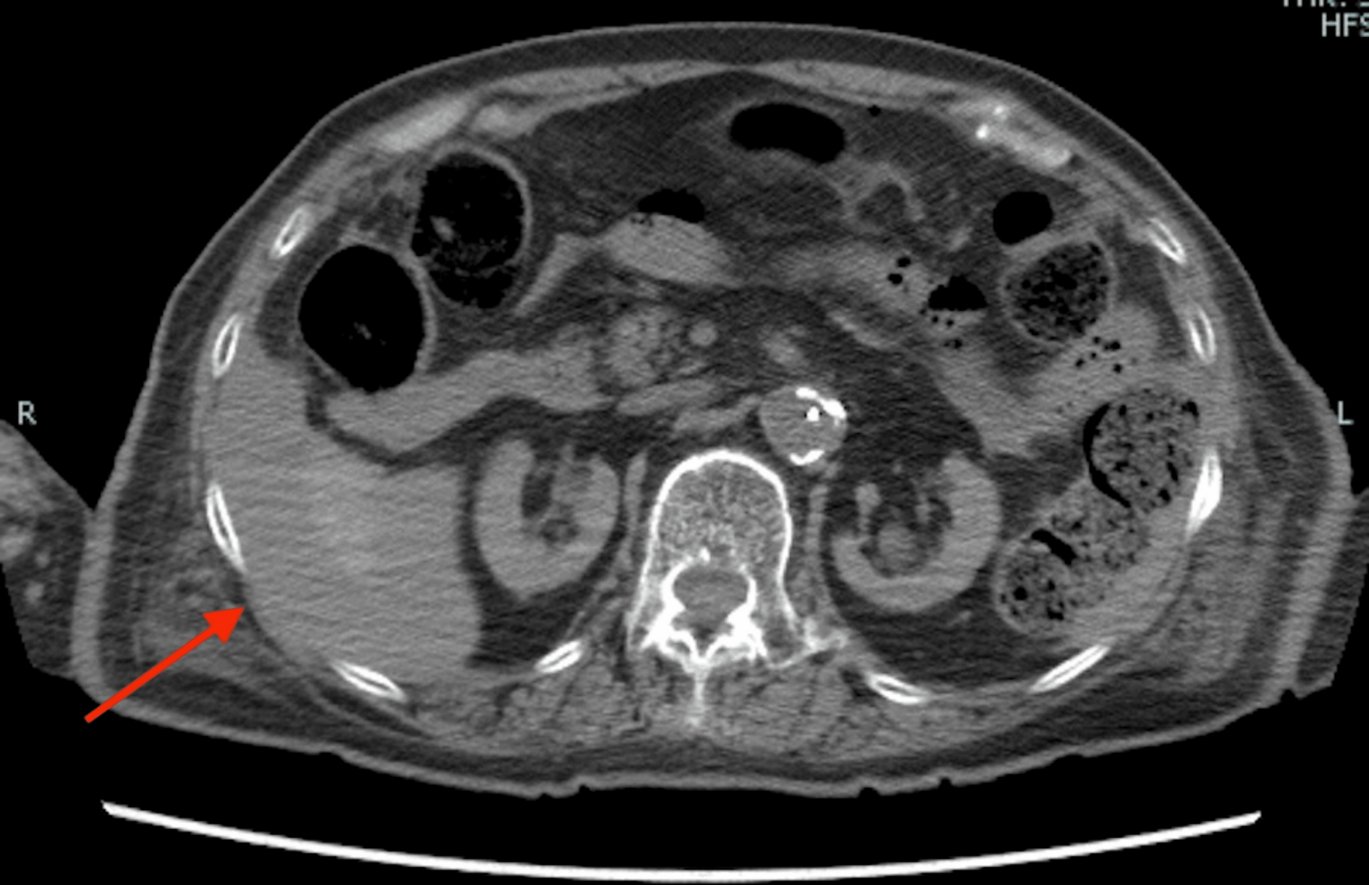
CT image (abdomen) CT scan showing hemoperitoneum with evidence of hepatic lacerations.

**Figure 3:**
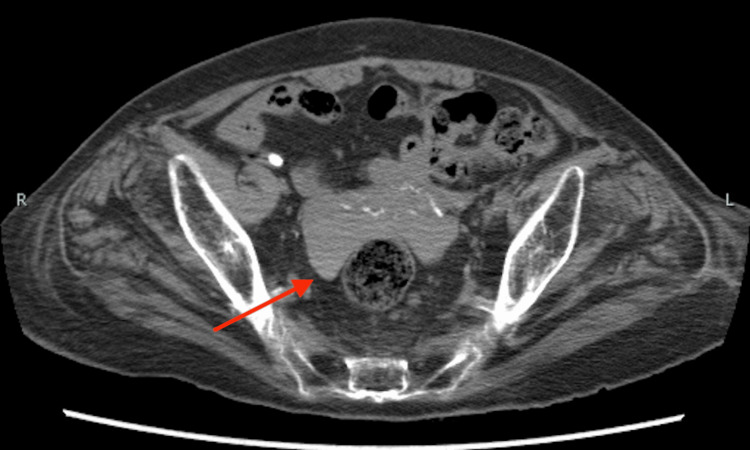
CT image (pelvic cavity) CT image demonstrating blood accumulation in the pelvic cavity, suggestive of intra-abdominal hemorrhage.

Postmortem CT findings revealed extensive bloody ascites in the abdomen and pelvis. Figure 1 shows findings suggestive of lacerations in the liver and spleen, while Figure 2 displays hemoperitoneum likely due to liver injury, and Figure 3 indicates bleeding within the pelvic cavity. These observations are consistent with blunt abdominal trauma.

Postmortem examination findings

Although a formal autopsy was not performed, a limited external examination after death revealed a faint seatbelt mark in the upper abdomen that had not been apparent during the initial evaluation. This finding was consistent with blunt abdominal trauma attributed to seatbelt syndrome.

## Discussion

REBOA and ET

REBOA and ET are aggressive interventions used in traumatic hemorrhagic shock and cardiac arrest.

Although REBOA can provide temporary hemorrhage control, its impact on overall survival remains uncertain. A national trauma registry study reported a mortality rate of 35.7% in the REBOA group compared to 18.9% in the non-REBOA group. However, this difference likely reflects the greater injury severity in the REBOA group rather than a direct effect of REBOA itself [6]. A recent systematic review found no significant difference in survival between REBOA and non-REBOA patients after adjusting for injury severity, highlighting the need for strict patient selection criteria [6].

Similarly, ET has limited efficacy, particularly in elderly patients with blunt trauma. The survival rate for ET in blunt trauma is approximately 1.4% [7]. Studies have reported near-zero survival in patients over 57 years of age, emphasizing the need for extreme caution when considering ET in this population.

Geriatric traumatic OHCA and accident prevention

Traumatic OHCA has a historically dismal prognosis, with a survival-to-discharge rate of approximately 2% [1]. However, recent studies indicate that survival can reach 5-8% in specific scenarios, such as in military settings or regions with highly advanced prehospital care systems [2]. This emphasizes the importance of rapid intervention and proper patient selection.

A nationwide Japanese study analyzing injured patients over a 10-year period reported a significant decrease in in-hospital mortality among elderly trauma patients [8]. In particular, mortality among severely injured elderly patients (Injury Severity Score (ISS) ≥16) declined from 26.1% to 14.5%, likely due to improvements in trauma care.

Given these challenges, primary and secondary prevention strategies are paramount. Seatbelt use is one of the most effective interventions, reducing fatality risk by up to 50% for front-seat occupants [5]. For rear-seat passengers, the risk reduction varies between 25% and 75%, depending on crash circumstances and proper belt use.

Seatbelt syndrome and risk factors

Seatbelts are essential for reducing injury severity, but improper fit can lead to "seatbelt syndrome," which involves injuries to the abdomen, spine, and vascular structures [4,9]. Multiple case reports have documented severe intra-abdominal injuries associated with seatbelt misuse. For example, Nigam and Nigam described a case in which a lap belt led to extensive hepatic and intestinal trauma, emphasizing the importance of improved restraint systems for high-risk populations [9]. Notably, two-point lap belts concentrate force on the abdominal organs and pose a particular danger to children and small adults under 140 cm [5]. Standard seatbelts are designed for individuals 140 cm or taller, and improper belt positioning in shorter occupants increases the risk of abdominal injuries associated with seatbelt syndrome.

In our case, the patient's short stature (140 cm) and wheelchair-dependent posture likely resulted in inadequate belt positioning, contributing to her fatal injuries. Research has highlighted that individuals with atypical body configurations, including wheelchair users, are disproportionately affected by seatbelt misalignment [10]. Improved restraint systems that can be adapted to diverse body types and mobility needs are crucial for reducing injury risk. Notably, the vehicle involved in this collision was manufactured in 2008, just before Japan's mandatory regulation requiring three-point seatbelts in rear seats took effect, leaving only a two-point lap belt available for this wheelchair-dependent occupant.

Additionally, the "submarine effect," wherein an occupant slides beneath the lap belt, has been documented in case studies and is another mechanism of severe injury [11,12]. These reports underscore the critical importance of proper restraint geometry and positioning.

Wheelchair users and seatbelt syndrome

**Figure 4:**
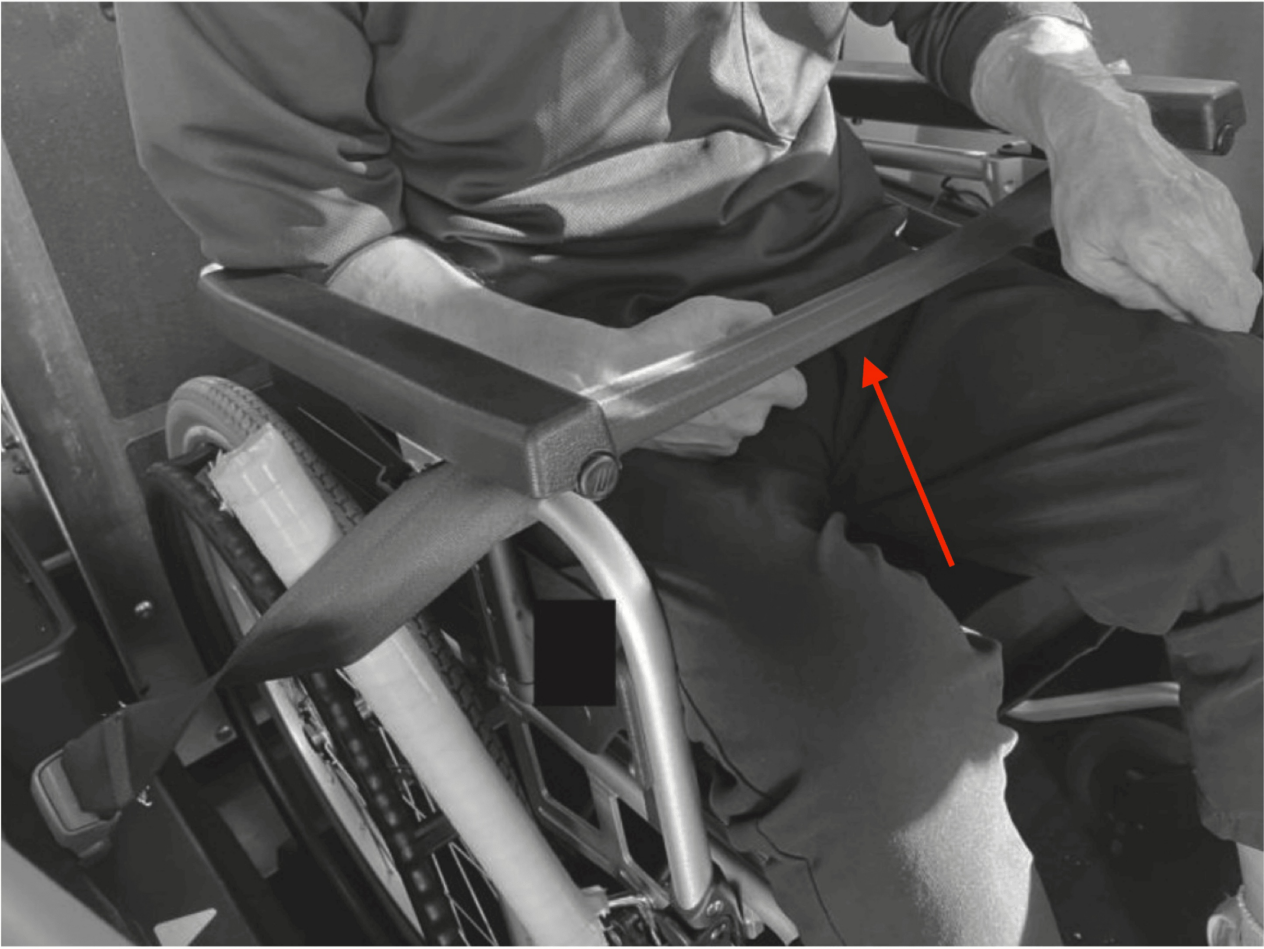
Example of a lap belt restraint for a wheelchair user in a kei car The wheelchair's armrest interferes with the belt, preventing it from being properly secured at the navel. As shown in this photograph, depending on the wheelchair user's body type and seating position, the belt may not fit snugly and thus may be ineffective. Adapted from Kuwahara and Hitosugi [12], with permission from the authors and publishers.

Wheelchair users face disproportionately high risks in vehicle collisions due to inadequate seatbelt fit and limited restraint options [13]. Figure 4 provides an example of how a lap belt may be positioned in a small adapted vehicle for wheelchair users, highlighting potential fit issues. A Japanese regional study found that wheelchair users accounted for 7.8% of fatal vehicle-occupant crashes in Shiga Prefecture from 2020 to 2022, nearly doubling from previous years [14]. However, this statistic is not based on a nationwide dataset, and further research is needed to assess trends at the national level.

Despite the clear risks, comprehensive data on injury mechanisms and outcomes for wheelchair users remain scarce. A recent study in Japan emphasized the need for a nationwide registry of wheelchair-occupant injuries and fatalities, which could facilitate better policy-making and more effective safety improvements [14].

Engineering analyses demonstrate that standard vehicle seatbelts are often inadequate for wheelchair users, as suboptimal positioning significantly increases the risk of severe injuries [15]. As shown in this photograph (Figure 4), depending on the wheelchair user's body type and seating position, the belt may not fit snugly and thus may be ineffective. Although the WHO has emphasized the importance of occupant restraints for all passengers, it has not issued specific guidelines for wheelchair restraint systems [10]. In the absence of such guidance, international safety standards, such as ISO 10542, recommend dedicated wheelchair tiedown and occupant restraint systems (WTORS) to improve safety for wheelchair users. Implementing WTORS could markedly reduce injury in this vulnerable population. Additionally, ensuring that transport personnel and caregivers are properly trained in seatbelt positioning is essential to minimizing preventable fatalities.

## Conclusions

This case illustrates how the convergence of multiple risk factors, namely, advanced age, wheelchair dependence, and short stature, can lead to catastrophic injuries even during relatively low-speed collisions. While REBOA may offer transient hemodynamic stabilization, prolonged full occlusion carries a substantial ischemic burden. Similarly, ET in geriatric blunt trauma has shown very limited efficacy, emphasizing the need for stringent patient selection. The concept of "seatbelt syndrome" further underscores how improperly fitted restraints can concentrate force on the abdomen or spine, thereby heightening the risk of severe injury.

These findings highlight the importance of developing specialized restraint systems, such as three-point belts and pelvic harnesses, tailored to wheelchair users and smaller-framed elderly individuals, as well as the critical role of education and policy initiatives for caregivers and drivers.

It is important to acknowledge that this report is based on a single case and may not be broadly generalizable. Moreover, current data regarding the efficacy of REBOA and ET in older adults, particularly those traveling in wheelchairs, remain sparse, underscoring the need for larger-scale research. Nevertheless, given the growing population of elderly individuals who rely on wheelchairs, a comprehensive, patient-centered approach to preventing seatbelt syndrome and improving trauma care in this vulnerable group is becoming increasingly imperative.
